# Eosinophil and lymphocyte counts predict bevacizumab response and survival in recurrent glioblastoma

**DOI:** 10.1093/noajnl/vdaa031

**Published:** 2020-03-11

**Authors:** Eugene J Vaios, Sebastian F Winter, Alona Muzikansky, Brian V Nahed, Jorg Dietrich

**Affiliations:** 1 Harvard Medical School, Boston, Massachusetts, USA; 2 Department of Biostatistics, Massachusetts General Hospital, Boston, Massachusetts, USA; 3 Department of Neurosurgery, Charité – Universitätsmedizin, Berlin, Germany; 4 Department of Neurology, Division of Neuro-Oncology, Massachusetts General Hospital, Boston, Massachusetts, USA; 5 Department of Neurosurgery, Massachusetts General Hospital, Boston, Massachusetts, USA

**Keywords:** bevacizumab, biomarkers, blood cell counts, clinical outcomes, glioblastoma

## Abstract

**Background:**

There is a lack of biomarkers to identify glioblastoma (GBM) patients who may benefit from specific salvage therapies, such as the anti-angiogenic agent bevacizumab. We hypothesized that circulating blood counts may serve as biomarkers for treatment response and clinical outcomes.

**Methods:**

Complete blood counts, clinical data, and radiographic information were collected retrospectively from 84 recurrent GBM patients receiving bevacizumab (10 mg/kg every 2 weeks). Significant biomarkers were categorized into quartiles and the association with clinical outcomes was assessed using the Kaplan–Meier method.

**Results:**

The median treatment duration and survival on bevacizumab (OS-A) was 88 and 192 days, respectively. On multivariate analysis, *MGMT* promoter methylation (hazard ratio [HR] 0.504, *P* = .031), increases in red blood cells (HR 0.496, *P* = .035), and increases in eosinophils (HR 0.048, *P* = .054) during treatment predicted improved OS-A. Patients in the first and fourth quartiles of eosinophil changes had a 12-month survival probability of 5.6% and 41.2% (*P* < .0001), respectively. Treatment response was associated with increases in eosinophil counts (*P* = .009) and improved progression-free survival (*P* = .013). On multivariate analysis, increases in lymphocyte counts among responders predicted improved OS-A (HR 0.389, *P* = .044). Responders in the first and fourth quartiles of lymphocyte changes had a 12-month survival probability of 0% and 44.4% (*P* = .019), respectively. Changes in platelet counts differed before and after radiographic response (*P* = .014).

**Conclusions:**

Changes in circulating eosinophil, lymphocyte, and platelet counts may predict treatment response and clinical outcomes in patients with recurrent GBM receiving bevacizumab.

**Key Points**

- Changes in eosinophil and lymphocyte counts predict bevacizumab response and survival.
- Changes in platelet counts correlate with radiographic response to bevacizumab.
- Circulating blood counts may reflect changes in tumor biology and the host immune response.

**Importance of the Study**

Bevacizumab remains one of the few FDA approved and most commonly prescribed salvage therapies for recurrent GBM. However, the reduction in tumor size following therapy is transient and it is unclear which patients are most likely to benefit from treatment. Additionally, radiographic assessment of response to bevacizumab is undermined by alterations in contrast permeability, creating challenges for monitoring tumor progression. The identification of a blood biomarker to guide patient management remains an active area of interest. To our knowledge, this is the first study to report an association between changes in peripheral blood counts and clinical outcomes in recurrent GBM treated with bevacizumab. These findings may set the stage for future investigations to assess whether these circulating biomarkers are indicative of the complex interactions between the tumor microenvironment and the patient’s antitumor immune response in the setting of bevacizumab therapy.

Glioblastoma (GBM) is the most common and aggressive primary brain tumor in adults. The standard of care includes maximal safe resection, followed by chemoradiation and adjuvant temozolomide (TMZ). GBM ultimately recurs and has proven resistant to available treatment modalities. Despite advances in surgery, antineoplastic agents, and radiation therapies, the prognosis remains poor with a median survival of less than 18 months following surgery and chemoradiation.^1^

Endothelial proliferation is among the diagnostic hallmarks of GBM and angiogenesis plays a critical role in the progression and clinical behavior of these tumors. Inhibition of the complex process of neovascularization, which involves tissue remodeling, growth, and destruction, has emerged as a complement therapy to standard treatment regimens. Bevacizumab, a humanized monoclonal antibody against vascular endothelial growth factor A (VEGF-A), was the first approved antiangiogenic drug after clinical trials found that it significantly improved radiographic response rates when combined with other cytotoxic agents.^2,3^ However, not all patients show response to bevacizumab treatment and in responders, radiographic reduction in tumor size may only be transient, likely the result of upregulation of VEGF-independent pathways.^4^ Despite the clinical utility of antiangiogenic therapies, no surrogate marker of treatment response has been validated in cancer patients to guide clinical management.

Various reasons may account for the generally poor response to salvage therapy, including the highly invasive behavior of glioma cells within the brain parenchyma, the existing challenges of sufficient drug delivery across the blood-brain barrier, and the genetic heterogeneity of GBM with the rapid emergence of resistance mechanisms.^5,6^ Currently, there are no biomarkers for the identification of alternative growth pathways in the setting of resistance to antiangiogenic therapy. Additionally, accurate assessment of radiographic response is undermined by alterations in contrast permeability during treatment, creating challenges in quantifying response and monitoring tumor progression during active therapy.^7^ These insights have elevated the importance of noninvasive biomarkers for treatment response and clinical outcomes that potentially would allow for real-time selection and adjustment of personalized therapies.

Bevacizumab improves progression-free survival (PFS) in GBM patients with recurrent disease; however, response varies, and disease ultimately progresses. Despite ongoing efforts to identify imaging-based biomarkers, there is no predictive marker for patients who will maximally benefit from bevacizumab. The identification of a noninvasive, peripheral biomarker of treatment response and overall outcomes could improve patient management and potentially alter current dosing protocols that do not account for interpatient variability in resistance mechanisms. We previously demonstrated that bone marrow toxicity, reflected by changes in circulating white blood cell counts, was predictive of overall survival (OS) in patients with *newly diagnosed* GBM treated with standard chemoradiation.^8^ We here examined the question of whether changes in circulating blood counts in patients treated with bevacizumab for *recurrent* disease could serve as a potential biomarker of treatment response and clinical outcomes.

## Materials and Methods

We conducted a retrospective analysis of clinical and demographic data from patients who previously underwent surgery and treatment for primary GBM at the Massachusetts General Hospital (MGH) between 2004 and 2015. Patient data were obtained from an MGH institutional database. This study was approved by the MGH institutional review board for all activities.

### Eligibility

All patients were treated at the MGH and met the following eligibility criteria: newly diagnosed with GBM (WHO grade IV) between November 19, 2004, and January 6, 2015; 18 years of age or older at the time of diagnosis; surgical biopsy and resection after initial presentation; treatment with standard chemoradiation including at least 2 cycles of monthly adjuvant TMZ, followed by at least 2 cycles of salvage therapy with single-agent bevacizumab for recurrent disease. Bevacizumab was administered intravenously at a dose of 10 mg/kg of body weight every 2 weeks. Patients who did not complete at least 2 cycles of TMZ therapy and bevacizumab monotherapy were excluded from the analysis.

### Variables

Descriptive information, including age, gender, and steroid use, was collected. Steroid use was defined as exposure to steroids (eg, dexamethasone) at any given time during the course of bevacizumab treatment. Genetic information including chromosomal abnormalities, point mutations, and gene methylation was recorded. This included known prognostic markers for gliomas such as epidermal growth factor receptor (*EGFR*) amplification and *MGMT* promoter methylation. The genetic characteristics of the sample are reported as the percentage of patients for whom that genetic mutation was present. Absolute peripheral platelet, red blood cell, white blood cell, lymphocyte, neutrophil, monocyte, eosinophil, and basophil count measurements were recorded at discrete time points during the course of treatment. Time points included before surgery, after surgery, before chemoradiation, at each monthly TMZ treatment cycle, before initiation of bevacizumab, and at regular intervals across the duration of bevacizumab treatment. Clinical outcome measures included OS and PFS. Treatment response was defined as a quantifiable decrease greater than 20–25% of the contrast-enhancing mass on magnetic resonance imaging.

### Statistics

The primary outcome measure was the OS. Time intervals for this endpoint were calculated from diagnosis to death or last date known to be alive for those who were censored. OS-A used the length of time from initiation of bevacizumab to death or last date known to be alive for those who were censored. The secondary outcome measure was PFS-A. Time intervals for this endpoint were calculated from the date of bevacizumab initiation to the time of the first progression based on radiology report or clinician notes indicating a switch in therapy or last date known to be progression free for censored patients. The effect of changes in peripheral blood counts on clinical outcomes was assessed during the interval between baseline measurement (initiation of bevacizumab therapy) and either the time of response or first progression while on treatment. All blood counts were gathered closest to the time of event, but no longer than 2 weeks before or after.

Genetic variables known to be strong prognostic markers were tested for association with clinical outcomes using logistic regression or Pearson’s chi-square test.* IDH* mutation and other genetic markers (*P53*, *PTEN*, *PIK3CA*, *Beta-Catenin,PDGFR*, and *MET*) were present in fewer than 10 patients and excluded from this analysis. Spearman or Pearson correlation coefficients were estimated to measure the relation between continuous or ordinal baseline demographic variables and clinical outcome measures. Wilcoxon rank-sum tests and Kruskal–Wallis tests by ranks were used to examine differences in mean blood count changes between groups of patients stratified by response to bevacizumab. On subgroup analysis, changes in blood counts before and after response were compared using a paired samples *t*-test. Univariate and multivariate Cox proportional hazards models were used to evaluate variables for association with OS, OS-A, and PFS-A. Variables were chosen for multivariate analysis using the backward selection method based on the statistical significance on univariate analysis. All reported *P*-values were two-sided, and statistical significance was considered as *P* < .05. On subgroup analysis, significant biomarkers were subsequently categorized into quartiles and the Kaplan–Meier method was used to test differences between these groups in clinical outcomes.

## Results

### Descriptive Data Analysis

In total, 84 patients diagnosed with GBM were included in this analysis. The median age at the time of diagnosis was 58 years; 52 patients (62%) were men and 32 (38%) were women. Two patients (2.4%) were alive at the time of data cutoff for analysis. The median OS for the entire group was 733 days. Sixty-four (76%) patients completed at least 6 monthly cycles of adjuvant TMZ and the median duration of bevacizumab monotherapy was 88 days. On molecular genetic analysis, 40 (47.6%) patients had an *EGFR* amplification and 28 (33.3%) patients had *MGMT* promoter methylation (Supplementary Table S1). Changes in blood counts from the start of bevacizumab treatment to the first progression among responders and nonresponders are listed in Supplementary Table S3. Changes in peripheral blood counts before and after evidence of radiographic response are listed in Supplementary Table S4.

### Biomarkers of Clinical Outcomes Among Patients Receiving Bevacizumab

**Figure 1.**
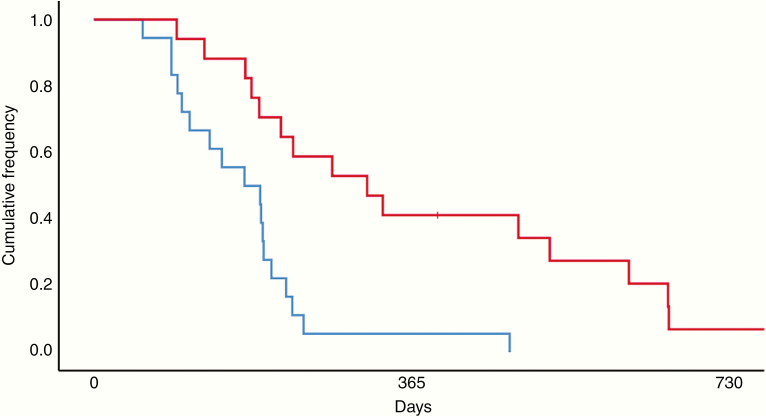
Kaplan–Meier survival curve for patients stratified by changes in eosinophil counts. The 12-month survival rate for patients in the first (blue) and fourth (red) quartiles of changes in eosinophil counts during therapy was 5.6% and 41.2%, respectively. This corresponded to a median OS-A of 173 and 314 days (*P* < .0001) after starting bevacizumab. Patients in the first and fourth quartiles had a median change in eosinophil counts of −80% and 260% from baseline, respectively (OS-A = survival time from bevacizumab initiation to death).

Consistent with the literature, *MGMT* promoter methylation was associated with improved OS (*P* = .001; Supplementary Table S2).^9^ In a univariate analysis, *MGMT* status (*P* = .004) and changes in platelet (*P* = .017), red blood cell (*P* = .009), and eosinophil (*P* = .006) counts were significantly associated with OS-A (Table 1). On multivariate analysis, *MGMT* promoter methylation (*P* = .031) and increases in red blood cell counts (*P* = .035) remained significantly associated with improved OS-A. The association between increases in eosinophil counts and improved OS-A (*P* = .054) trended toward significance (Table 1). On subgroup analysis, patients were stratified based on the magnitude of change in eosinophil counts. The Kaplan–Meier estimated 12-month survival rate was 5.6% and 41.2% for patients in the first and fourth quartiles (*P* < .0001; Figure 1), respectively. This corresponded to a median OS-A of 173 and 314 days (*P* < .0001), respectively. Baseline eosinophil counts in the first quartile (M_1_ = 0.1172, SD_1_ = 0.0972) were elevated compared to those in the fourth quartile (M_4_ = 0.0465, SD_4_ = 0.0522; *P* = .012). At the conclusion of treatment, eosinophil counts in the first quartile had declined and were significantly lower (M_1_ = 0.0372, SD_1_ = 0.0443) than those in the fourth quartile (M_4_ = 0.1853, SD_4_ = 0.1388; *P* < .0001). We did not observe any significant predictors of PFS-A on univariate analysis (Supplementary Table S5).

**Table 1.** Univariate and Multivariate Analysis for OS-A

Univariate Analysis			
Covariate	HR	95% CI	P*
Gender			
Male	1.121	0.715–1.757	.618
Female	—	—	—
Age	1.006	0.986–1.026	.584
Genetic mutations			
*EGFR*	0.778	0.491–1.234	.286
*MGMT*	0.458	0.271–0.774	.004
Blood count changes^a^			
Platelets	0.995	0.991–0.999	.017
Red blood cells	0.533	0.332–0.857	.009
White blood cells	1.006	0.929–1.090	.877
Neutrophils	1.023	0.942–1.110	.590
Lymphocytes	0.697	0.405–1.200	.192
Monocytes	0.361	0.103–1.268	.112
Eosinophils	0.018	0.001–0.319	.006
Basophils	0.033	0.000–76.656	.388
Steroids			
Used	1.413	0.832–2.401	.201
Not used	—	—	—
Multivariate Analysis			
Covariate	HR	95% CI	P*
*MGMT*	0.504	0.270–0.941	.031
Platelets	0.997	0.993–1.002	.209
Red blood cells	0.496	0.258–0.953	.035
Eosinophils	0.048	0.002–1.057	.054

Association of OS-A with gender, age, *EGFR* amplification, *MGMT* promoter methylation, changes in blood counts during treatment, and steroid use. Significant variables in the univariate analysis were included in the multivariate analysis. Hazard ratios with 95% confidence intervals and statistical significance are shown (OS-A = survival time from bevacizumab initiation to death).

^a^Blood count changes are those that occurred throughout therapy.

*Based on the log-rank test.

### Biomarkers of Clinical Outcomes Among Bevacizumab Responders

**Figure 2.**
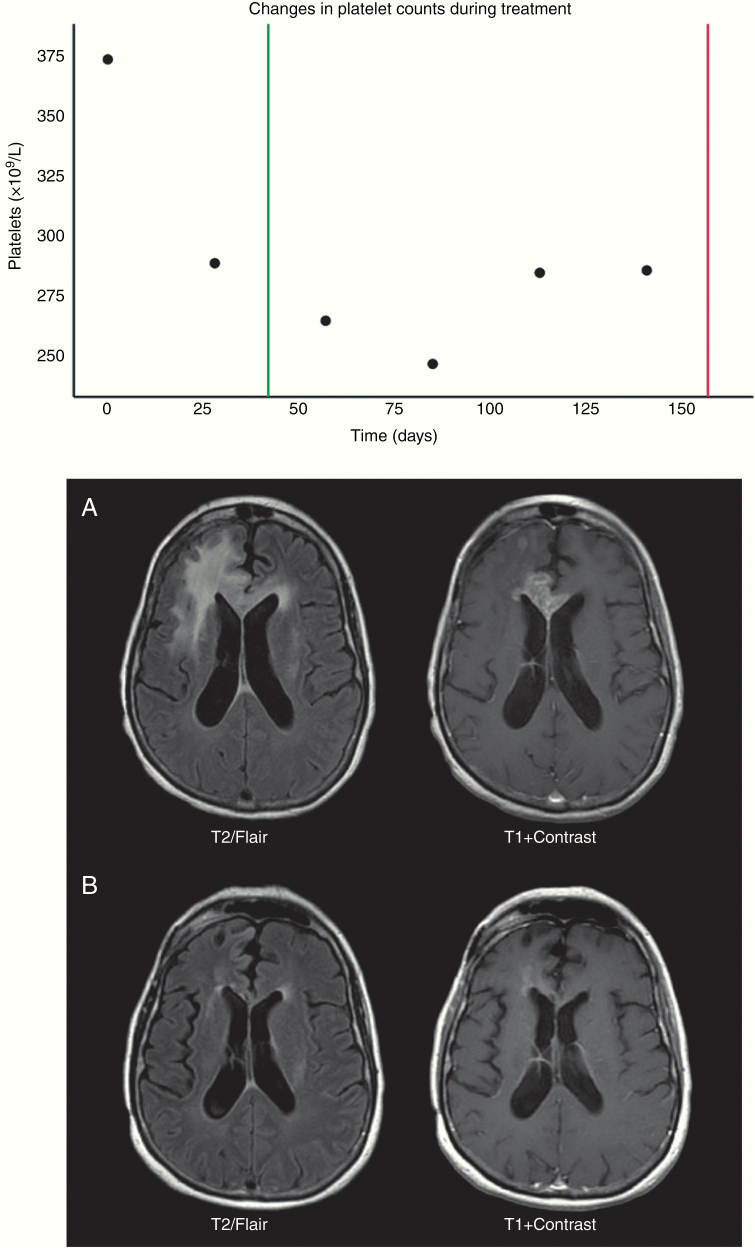
Changes in platelet counts correlate with bevacizumab response. (A) A representative patient’s platelet counts over the duration of bevacizumab therapy. Response and progression dates denoted in green and red, respectively. Platelet count decreases correspond with a response to treatment, with increases preceding evidence of progressive disease. (B) T2/FLAIR and T1-postcontrast images at the time of bevacizumab initiation (a) and at the time of response to bevacizumab (b).

In total, 48 (57%) patients showed radiographic evidence of treatment response to bevacizumab. Treatment response was defined as a quantifiable decrease greater than 20–25% of the contrast-enhancing mass on radiographic imaging. Bevacizumab responders had significantly improved PFS-A compared to nonresponders (*P* = .013; Table 2). There was no significant difference in gender, age, duration of adjuvant TMZ in the newly diagnosed setting, or steroid use between groups. Increases in eosinophil counts were associated with treatment response (*P* = .009; Table 2). Among responders, absolute changes in platelet counts differed before and after radiographic response to therapy (*P* = .014), with decreases (M_1_ = −21.52, SD_1_ = 35.62) and increases (M_2_ = 8.73, SD_2_ = 52.14) occurring before and after radiographic response, respectively (Supplementary Table S4 and Supplementary Table S6; Figure 2). In a univariate analysis, *MGMT* status (*P* = .040) and changes in platelet (*P* = .039), red blood cell (*P* = .023), and lymphocyte (*P* = .047) counts prior to radiographic response were significantly associated with OS-A (Table 3). On multivariate analysis, increases in lymphocyte counts remained significantly associated with improved OS-A (*P* = .044), even after controlling for *MGMT* promoter methylation and other markers significant on univariate analysis (Table 3).

**Table 2.** Comparison of Patient Characteristics Between Bevacizumab Responders and Nonresponders

Characteristic	Responder (*n* = 48)^a^	Nonresponder (*n* = 36)	P*
Gender			
Male	31 (65%)	21 (58%)	.559
Female	17 (35%)	15 (42%)	
Age at diagnosis, years			
Median (range)	59 (26–73)	56 (40–85)	.926
6 Months adjuvant TMZ	38 (79%)	26 (72%)	.460
OS, days			
Median (range)	842 (247–3061)	681 (336–2311)	.076
OS-A, days			
Median (range)	200 (56–2093)	167 (41–984)	.073
PFS-A, days			
Median (range)	120 (21–876)	61 (16–797)	.013
Genetic mutations			
*EGFR*	20 (42%)	20 (56%)	.142
*MGMT*	19 (40%)	9 (25%)	.136
Blood count changes^b^			
Platelets (×10^9^/L)			
Median	−8.00	−29.00	.403
Range	−108.00 to 146.00	−229.00 to 163.00	
Red blood cells (×10^12^/L)			
Median	0.15	0.145	1.000
Range	−1.37 to 1.38	−0.84 to 1.26	
White blood cells (×10^9^/L)			
Median	−0.17	−0.10	
Range	−5.60 to 5.70	−6.90 to 11.80	.296
Neutrophils (×10^9^/L)			
Median	−0.44	−0.18	.260
Range	−6.50 to 5.47	−6.84 to 12.41	
Lymphocytes (×10^9^/L)			
Median	0.04	−0.08	.325
Range	−1.14 to 1.52	−0.99 to 1.65	
Monocytes (×10^9^/L)			
Median	0.04	−0.02	.078
Range	−0.28 to 0.47	−0.56 to 0.52	
Eosinophils (×10^9^/L)			
Median	0.03	−0.01	.009
Range	−0.07 to 0.52	−0.35 to 0.25	
Basophils (×10^9^/L)			
Median	0.01	0.00	.361
Range	−0.14 to 0.04	−0.07 to 0.06	
Steroid use	39 (81%)	26 (72%)	.328

Reported differences in gender, age, adjuvant temozolomide use, clinical outcomes, genetic mutations, changes in circulating blood counts, and steroid use between groups. All reported *P*-values calculated based on chi-square test or ANOVA (OS = survival time from diagnosis to death; OS-A = survival time from bevacizumab initiation to death; PFS-A = time from bevacizumab initiation to the first progression).

Values represent *n* (%) unless otherwise indicated.

^a^Response defined as a quantifiable decrease of at least 20–25% of the contrast-enhancing mass.

^b^Blood count changes over the entire duration of bevacizumab therapy.

*Based on the log-rank test.

**Table 3.** Univariate and Multivariate Analysis for OS-A Among Responders

Covariate	HR	95% CI	P*
Univariate Analysis			
Gender			
Male	1.252	0.682–2.300	.469
Female	—	—	—
Age	1.010	0.980–1.041	.518
Genetic mutations			
*EGFR*	0.718	0.388–1.329	.291
*MGMT*	0.486	0.245–0.966	.040
Blood count changes^a^			
Platelets	0.990	0.981–1.000	.039
Red blood cells	0.351	0.142–0.868	.023
White blood cells	1.058	0.925–1.210	.413
Neutrophils	1.075	0.913–1.264	.385
Lymphocytes	0.491	0.243–0.992	.047
Monocytes	1.743	0.255–11.896	.571
Eosinophils	0.004	0.000–2.927	.100
Basophils	0.001	0.000–60.514	.226
Steroids			
Used	2.024	0.912–4.493	.083
Not used	—	—	—
Multivariate Analysis			
*MGMT*	0.457	0.191–1.095	.079
Platelets	0.991	0.977–1.006	.222
Red blood cells	1.295	0.349–4.809	.699
Lymphocytes	0.389	0.155–0.977	.044

Association of OS-A with gender, age, *EGFR* amplification, *MGMT* promoter methylation, changes in blood counts, and steroid use. Significant variables in the univariate analysis were included in the multivariate analysis. Hazard ratios with 95% confidence intervals and statistical significance are shown (OS-A = survival time from bevacizumab initiation to death).

^a^Blood count changes are those that occurred prior to radiographic response.

*Based on the log-rank test.

**Figure 3.**
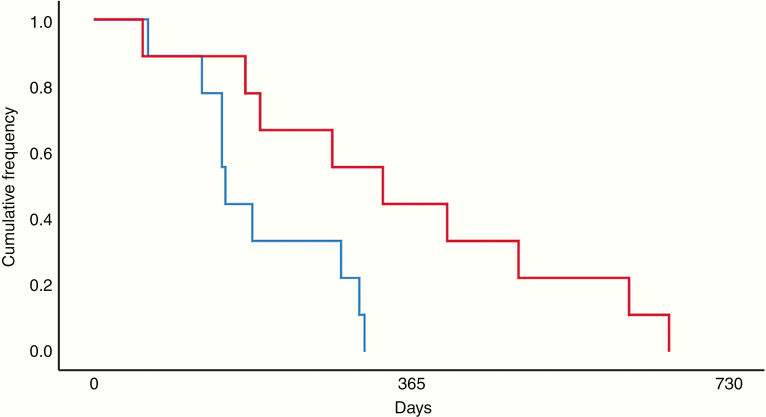
Kaplan–Meier survival curve for patients stratified by changes in lymphocyte counts. The 12-month survival rate for patients in the first (blue) and fourth (red) quartiles of changes in lymphocytes prior to radiographic response was 0% and 44.4%, respectively. This corresponded to a median OS-A of 151 and 332 days (*P* = .019), respectively. Patients in the first and fourth quartiles had a median change in lymphocyte counts of −52.27% and 88.89% from baseline, respectively (OS-A = survival time from bevacizumab initiation to death).

On subgroup analysis, responders were stratified based on the magnitude of change in lymphocyte counts. For patients in the first and fourth quartiles, the Kaplan–Meier 12-month survival rate was 0% and 44.4%, respectively. This corresponded to a median OS-A of 151 and 332 days (*P* = .019; Figure 3), respectively. Patients in the first quartile had significantly higher lymphocyte counts (M_1_ = 1.2344, SD_1_ = 0.4926) at baseline compared to patients in the fourth quartile (M_4_ = 0.690, SD_4_ = 0.2787; *P* = .011). At the time of radiographic response, lymphocyte counts were significantly lower among patients in the first quartile (M_1_ = 0.660, SD_1_ = 0.3945) relative to patients in the fourth quartile (M_4_ = 1.2933, SD_4_ = 0.4032; *P* = .004). There was no association between changes in blood counts and PFS-A among responders (Supplementary Table S7).

## Discussion

We observed that treatment-associated changes in eosinophil, lymphocyte, and platelet counts during bevacizumab therapy may predict treatment response and clinical outcomes in patients with recurrent GBM. Increases in eosinophil counts were significantly associated with improved OS-A and radiographic response to bevacizumab. Similarly, elevations in lymphocyte counts prior to radiographic response predicted improved OS-A. This association was maintained even after controlling for known prognostic markers, including *MGMT* promoter methylation and steroid use. There was no statistically significant difference in OS-A or changes in lymphocyte counts prior to radiographic response between steroid users and nonusers. Additionally, changes in platelet counts differed before and after evidence of response, with decreases preceding radiographic response. Finally, a significant association was observed between increases in red blood cell counts and improved OS-A, which may represent a surrogate marker for bone marrow function following cessation of TMZ.

The identification of a circulating biomarker for treatment response and clinical outcomes in patients receiving bevacizumab is an active area of investigation. Circulating VEGF, placental growth factor, collagen IV, stromal-cell-derived factor 1a, and interleukin-6 have been considered as potential predictors of treatment response and OS.^10^ Circulating endothelial cells (CECs) and progenitor cells (CPCs) have also emerged as potential biomarkers for treatment response given their elevated levels in cancer patients.^11–16^ It has been shown that anti-angiogenic therapy with bevacizumab decreases the number of viable CECs and CPCs in patients with colorectal cancer.^17^ Likewise, studies suggest that infiltrating myeloid cells derived from the bone marrow interact with the tumor microenvironment and initiate a signaling cascade that serves as a surrogate for hypoxia and necrosis, resulting in activation of VEGF-independent proangiogenic pathways.^18–22^

These circulating cell populations contribute to the cellular complexity within the brain tumor microenvironment and the cancer stem cell niche, modulating processes critical to tumor behavior such as angiogenesis and cancer cell invasiveness.^8,23,24^ This view has been supported by studies demonstrating that subsets of circulating cells preferentially home to tumors and are implicated in tumor angiogenesis and tumor progression, underscoring the critical interaction between the local and systemic tumor environment in overall tumor biology and patient outcomes.^25–28^ Changes in circulating blood counts may reflect alterations within the tumor microenvironment in response to therapy. The use of circulating blood counts as markers for clinical outcomes during anti-angiogenic therapy has been demonstrated by some groups, such as by Zhu et al.,^29^ who showed an association between changes in platelet counts and changes in circulating VEGF-C and soluble VEGFR3 in hepatocellular carcinoma patients treated with the anti-VEGF tyrosine kinase inhibitor sunitinib. Consistent with other studies, the authors also observed that changes in myeloid cells during anti-angiogenic treatment were significantly correlated with OS and PFS.^30^

Our institutional survival data for patients with increases in eosinophil and lymphocyte counts compare favorably with data from the original BRAIN trial, which reported a median survival of 9.2 months for patients receiving bevacizumab alone.^31^ The observed association between increases in peripheral eosinophil and lymphocyte counts and improved clinical outcomes may reflect changes in the local tumor environment and the patient’s antitumor immune response. The role of tumor-infiltrating lymphocytes and inflammatory markers in predicting outcomes in GBM patients has been well established.^32,33^ Previous studies demonstrate that T-lymphocytes, in particular, inhibit tumor cell proliferation and invasion via cytokine production and induction of tumor cell death, suggesting an interaction between the systemic and local tumor environment.^34^ CD8^+^ cytotoxic T-lymphocytes have been shown to play an important role in the host tumor-specific adaptive immune system response.^33^ Circulating composite markers such as the neutrophil-to-lymphocyte ratio and the platelet-to-lymphocyte ratio have also been explored as possible prognostic markers of significance in GBM.^32^ Consistent with the literature, we observed improved OS-A in patients with increases in lymphocyte counts prior to radiographic evidence of treatment response (Figure 3). This association with clinical outcomes may reflect the systemic immune response to GBM and correlate with underlying changes in the tumor microenvironment, including early activation of alternative growth pathways or tumor-mediated immunosuppression. The observed difference in baseline lymphocyte counts between patients in the first and fourth quartiles (Figure 3) underscores the importance of changes in peripheral cell counts as a marker of antitumor immune response and clinical outcomes. Future prospective studies are warranted to assess whether changes in lymphocyte counts during bevacizumab treatment predict clinical outcomes and correlate with changes in the tumor microenvironment.

Recent studies also suggest that eosinophils may promote the innate and adaptive antitumor immune response in GBM patients. In a cohort of 94 patients, Spina et al.^35^ identified a 45% increase in OS among patients with persistent eosinophil elevation following chemoradiation. Similarly, we observed improved OS-A and response to bevacizumab in patients with increases in eosinophil counts (Figure 1). Elevations in peripheral eosinophil counts may correlate with a more potent systemic antitumor immune response to GBM. Conversely, decreases in eosinophil counts may indicate an impaired immune response or development of tumor-mediated resistance pathways. GBM resistance to standard therapy results, in part, from the recruitment of T-regulatory cells and myeloid-suppressor cells that obstruct the antitumor immune response by promoting an immunosuppressive microenvironment. In patients with atopic disease, characterized by eosinophilia and impaired antigenic tolerance, authors observe improved survival with GBM, potentially resulting from the enhanced ability to overcome the immunosuppressive tumor microenvironment.^36^ Consistent with this observation, a clinical trial reported improved outcomes in patients with tissue eosinophilia following postoperative treatment using IL-2.^37^ Similarly, other investigators identified improved survival in patients with elevated serum CD23 and IgE levels, suggesting that eosinophils may play an important role in mediating the host antitumor response to GBM.^38,39^ The function of eosinophils, including the production of cytotoxic granules, neuromediators, pro-inflammatory cytokines, and angiogenic factors, provides a biological basis for these observations. Changes in peripheral eosinophil counts may therefore reflect the immunologic response to GBM during bevacizumab treatment and correlate with interactions between the local and systemic immune environments. The observed difference in baseline eosinophil counts between patients in the first and fourth quartiles (Figure 1) suggests that the temporal change in peripheral cell counts represents a more robust marker of treatment response and outcomes. Prospective investigations are needed to determine if changes in peripheral eosinophil counts during bevacizumab treatment predict survival and treatment response in patients with recurrent GBM.

Finally, our analysis identified that changes in circulating platelet counts correlate with a radiographic response to treatment (Figure 2). This observation may reflect changes in the local tumor microenvironment and tumor–platelet interactions. The role of platelets in promoting tumor growth and angiogenesis has been demonstrated in multiple studies. Growing tumors secrete thrombopoietic factors that result in thrombocytosis, which has been associated with worse clinical outcomes in several malignant tumors including GBM.^40^ Other work has shown that platelets enable tumor growth and metastasis by interfering with immunoregulatory mechanisms.^41–44^ Additionally, platelets can stimulate tumor angiogenesis via secretion of VEGF, PDGF, and tissue factor, promoting tumor adhesion and vessel hyperpermeability.^45–48^ Work by Boonyawan et al.^49^ recently identified increases in platelet counts following chemoradiation as a marker for worse OS in GBM, even after controlling for *MGMT* promoter methylation and performance status.^50^ These studies suggest that platelets play an important role in tumor growth, and changes in peripheral counts may reflect local changes in the tumor microenvironment in response to therapy.

Despite this study’s robust findings, the analysis is limited by its retrospective nature and modest sample size. Due to a lack of statistical power, we were unable to assess the association between *IDH* mutation and clinical outcomes. However, consistent with the literature, we previously reported a significant association between *IDH* mutation and improved OS in patients with newly diagnosed GBM.^8^ Additionally, due to variability in bevacizumab treatment duration and sample size limitations, we were unable to ascertain the timing of peak changes in hematological parameters. Further studies are necessary to investigate the role of *IDH* mutation in predicting bevacizumab response and to determine the timing of maximal changes in hematologic parameters. Other biomarkers such as blood pressure changes may also be relevant to clinical outcomes in patients exposed to bevacizumab and could be evaluated in the context of changes in circulating blood cells in future prospective studies.^51^ Given the association between peripheral blood counts and clinical outcomes, investigators should also determine whether changes in circulating hematopoietic progenitor cells predict bevacizumab response in patients with recurrent GBM.

## Conclusions

The observed association between peripheral blood counts and clinical outcomes may reflect changes in the microvascular niche and the complex interactions between the tumor microenvironment and antitumor immune response. Future prospective studies are warranted to assess whether circulating eosinophil, lymphocyte, and platelet counts can serve as potential biomarkers for treatment response and overall clinical outcomes in patients with recurrent GBM treated with bevacizumab.

## Supplementary Material

vdaa031_suppl_Supplementary_Tables

## Notes

**Authorship Statement**. (I) Conception and design: E.J.V. and J.D.; (II) Administrative support: J.D.; (III) Provision of study materials or patients: J.D.; (IV) Collection and assembly of data: E.J.V.; (V) Data analysis and interpretation: All authors; (VI) Manuscript writing: All authors; (VII) Final approval of manuscript: All authors.
