# Supplementary material for: Eosinophil and lymphocyte counts predict bevacizumab response and survival in recurrent glioblastoma
Source: Neurooncol Adv. 2020 Mar 11;2(1):vdaa031. doi: 10.1093/noajnl/vdaa031 (PMC7212859; doi:10.1093/noajnl/vdaa031)
Supplement: vdaa031_suppl_Supplementary_Tables [file vdaa031_suppl_supplementary_tables.docx]

**Supplementary material for Vaios *et al*. manuscript**

| Characteristic | Value |
| --- | --- |
| Gender   Male   Female | 52 (62%)  32 (38%) |
| Age at diagnosis, years   Median (Range) | 58 (26–85) |
| 6 Months adjuvant TMZ | 64 (76%) |
| PFS, days  Median (Range) | 347 (160-1595) |
| PFS-A, days   Median (Range) | 88 (16-876) |
| OS, days   Median (Range) | 733 (247-3061) |
| OS-A, days   Median (Range) | 192 (41-2093) |
| Genetic mutations  *EGFR*  *MGMT* | 40 (47.6%)  28 (33.3%) |
| Steroid use | 65 (77.4%) |
| Deceased | 82 (97.6%) |

Values represent n (%) unless otherwise indicated

**Supplementary table 1: Summary of patient characteristics.** Descriptive analysis of patient characteristics, including gender, age, adjuvant temozolomide use, clinical outcomes, genetic mutations, and steroid use. (OS-A = survival time from bevacizumab initiation to death; PFS-A = time from bevacizumab initiation to first progression)

|  | | | | |
| --- | --- | --- | --- | --- |
| Univariate Analysis | | | | |
| Covariate | HR | 95% CI | p Value* | |
| Gender   Male   Female | 1.173  - | 0.747-1.840  - | | 0.488  - |
| Age | 1.026 | 1.005-1.047 | | 0.017 |
| Genetic mutations  *EGFR*  *MGMT* | 0.970  0.422 | 0.605-1.555  0.256-0.695 | | 0.899  0.001 |

*Based on the log-rank test

**Supplementary table 2: Univariate analysis for OS.** Association of OS with gender, age, *EGFR* amplification, and *MGMT* promotor methylation. Hazard ratios with 95% confidence intervals and statistical significance shown.

|  | |
| --- | --- |
| Hematology | Value |
| Platelets (×10^9^/L)  Mean (SD)  Range | -15.91 (59.14)  -229.00-163.00 |
| Red blood cells (×10^12^/L)  Mean (SD)  Range | 0.14 (0.49)  -1.37-1.38 |
| White blood cells (×10^9^/L)  Mean (SD)  Range | -0.19 (2.98)  -6.90-11.80 |
| Neutrophils (×10^9^/L)  Mean (SD)  Range | -0.10 (3.09)  -6.84-12.41 |
| Lymphocytes (×10^9^/L)  Mean (SD)  Range | 0.04 (0.49)  -1.14-1.65 |
| Monocytes (×10^9^/L)  Mean (SD)  Range | 0.00 (0.23)  -0.56-0.52 |
| Eosinophils (×10^9^/L)  Mean (SD)  Range | 0.02 (0.11)  -0.35-0.52 |
| Basophils (×10^9^/L)  Mean (SD)  Range | 0.00 (0.03)  -0.14-0.06 |

**Supplementary table 3: Blood count changes during bevacizumab treatment.** Changes in absolute platelet, red blood cell, white blood cell, neutrophil, lymphocyte, monocyte, eosinophil, and basophil counts across entire cohort from initiation of bevacizumab to first progression.

|  | |
| --- | --- |
| Hematology | Value |
| Pre-Response | |
| Platelets (×10^9^/L)  Mean (SD)  Range | -21.52 (35.62)  -106.00-44.00 |
| Red blood cells (×10^12^/L)  Mean (SD)  Range | 0.09 (0.37)  -0.75-0.90 |
| White blood cells (×10^9^/L)  Mean (SD)  Range | -0.70 (2.48)  -7.21-5.10 |
| Neutrophils (×10^9^/L)  Mean (SD)  Range | -0.85 (2.22)  -6.77-4.06 |
| Lymphocytes (×10^9^/L)  Mean (SD)  Range | 0.04 (0.48)  -1.35-0.96 |
| Monocytes (×10^9^/L)  Mean (SD)  Range | 0.02 (0.20)  -0.41-0.66 |
| Eosinophils (×10^9^/L)  Mean (SD)  Range | 0.02 (0.06)  -0.08-0.19 |
| Basophils (×10^9^/L)  Mean (SD)  Range | 0.00 (0.02)  -0.12-0.03 |
| Post-Response | |
| Platelets (×10^9^/L)  Mean (SD)  Range | 8.73 (52.14)  -113.00-153.00 |
| Red blood cells (×10^12^/L)  Mean (SD)  Range | 0.01 (0.42)  -0.95-1.31 |
| White blood cells (×10^9^/L)  Mean (SD)  Range | 0.49 (2.30)  -4.10-6.40 |
| Neutrophils (×10^9^/L)  Mean (SD)  Range | 0.16 (2.19)  -4.47-5.67 |
| Lymphocytes (×10^9^/L)  Mean (SD)  Range | 0.03 (0.39)  -0.74-1.52 |
| Monocytes (×10^9^/L)  Mean (SD)  Range | 0.05 (0.23)  -0.61-0.50 |
| Eosinophils (×10^9^/L)  Mean (SD)  Range | 0.04 (0.13)  -0.12-0.62 |
| ­­­ Basophils (×10^9^/L)  Mean (SD)  Range | 0.00 (0.02)  -0.08-0.05 |

**Supplementary table 4: Blood count changes before and after radiographic response.** Changes in absolute platelet, red blood cell, white blood cell, neutrophil, lymphocyte, monocyte, eosinophil, and basophil counts among responders before and after radiographic response.

|  | | | | |
| --- | --- | --- | --- | --- |
| Univariate Analysis | | | | |
| Covariate | HR | 95% CI | p Value* | |
| Gender   Male   Female | 1.093  - | 0.701-1.704  - | | 0.694  - |
| Age | 0.999 | 0.979-1.019 | | 0.937 |
| Genetic mutations  *EGFR*  *MGMT* | 0.999  0.824 | 0.633-1.577  0.510-1.332 | | 0.996  0.430 |
| Blood count changes^a^  Platelets   Red blood cells   White blood cells   Neutrophils   Lymphocytes   Monocytes   Eosinophils   Basophils | 0.997  1.145  1.080  1.109  0.689  0.949  0.083  0.659 | 0.991-1.002  0.645-2.033  0.983-1.187  0.998-1.231  0.371-1.281  0.279-3.224  0.001-6.203  0.001-367.42 | | 0.209  0.643  0.108  0.054  0.239  0.933  0.258  0.897 |
| Steroids   Used   Not used | 1.232  - | 0.728-2.085  - | | 0.438  - |

*Based on the log-rank test

^a^ Blood count changes are those that occurred during first 2 months of therapy

**Supplementary table 5: Univariate analysis for PFS-A.** Association of PFS-A with gender, age, *EGFR* amplification, *MGMT* promotor methylation, changes in circulating blood counts over first 2 months of therapy, and steroid use. Hazard ratios with 95% confidence intervals and statistical significance shown. (PFS-A = time from bevacizumab initiation to first progression)

|  | | | | |
| --- | --- | --- | --- | --- |
| Covariate | t | df | p Value | |
| Platelets   Red blood cells   White blood cells   Neutrophils   Lymphocytes   Monocytes   Eosinophils   Basophils | -2.587  0.677  -1.823  -1.852  -0.037  -0.362  0.178  -0.342 | 37  36  37  30  29  28  26  27 | | 0.014  0.503  0.076  0.074  0.971  0.720  0.860  0.735 |

**Supplementary table 6: Comparison of blood count changes before and after radiographic response.** Changes in platelet, red blood cell, white blood cell, neutrophil, lymphocyte, monocyte, eosinophil, and basophil counts compared before and after radiographic evidence of response. All reported p values calculated based on paired samples t test (2-sided).

|  | | | | |
| --- | --- | --- | --- | --- |
| Univariate Analysis | | | | |
| Covariate | HR | 95% CI | p Value* | |
| Gender   Male  Female | 1.043  - | 0.571-1.905  - | 0.892  - | |
| Age | 1.013 | 0.983-1.044 | 0.402 | |
| Genetic mutations  *EGFR*  *MGMT* | 0.868  0.821 | 0.474-1.591  0.438-1.540 | 0.648  0.539 | |
| Blood count changes^a^  Platelets   Red blood cells   White blood cells   Neutrophils   Lymphocytes   Monocytes   Eosinophils   Basophils | 0.992  0.519  1.073  1.087  0.582  0.951  0.010  0.102 | 0.983-1.002  0.229-1.174  0.951-1.210  0.944-1.251  0.304-1.113  0.185-4.893  0.000-3.520  0.000-1997.89 | | 0.102  0.115  0.253  0.247  0.102  0.952  0.123  0.651 |

*Based on the log-rank test

^a^ Blood count changes are those that occurred prior to radiographic response

**Supplementary table 7: Univariate analysis for PFS-A among responders.** Association of PFS-A with gender, age, *EGFR* amplification, *MGMT* promotor methylation, changes in blood counts, and steroid use. Hazard ratios with 95% confidence intervals and statistical significance shown. (PFS-A = time from bevacizumab initiation to first progression)
